# Interaction of Ligands for PET with the Dopamine D3 Receptor: In Silico and In Vitro Methods

**DOI:** 10.3390/biom11040529

**Published:** 2021-04-02

**Authors:** Chia-Ju Hsieh, Aladdin Riad, Ji Youn Lee, Kristoffer Sahlholm, Kuiying Xu, Robert R. Luedtke, Robert H. Mach

**Affiliations:** 1 Division of Nuclear Medicine and Clinical Molecular Imaging, Department of Radiology, Perelman School of Medicine, University of Pennsylvania, Philadelphia, PA 19104, USA; chiahs@pennmedicine.upenn.edu (C.-J.H.); Aladdin.Riad@pennmedicine.upenn.edu (A.R.); JiYoun.Lee@Pennmedicine.upenn.edu (J.Y.L.); kuxu@pennmedicine.upenn.edu (K.X.); 2 Wallenberg Center for Molecular Medicine, Department of Integrative Medical Biology, Umea University, 901 87 Umea, Sweden; kristoffer.sahlholm@ki.se; 3 Department of Pharmacology and Neuroscience, University of North Texas Health Science Center-Fort Worth, Texas, TE 76107, USA; Robert.Luedtke@unthsc.edu

**Keywords:** Dopamine D3 receptor, [^18^F]Fallypride, [^18^F]Fluortriopride, docking, molecular dynamics

## Abstract

[^18^F]Fallypride and [^18^F]Fluortriopride (FTP) are two different PET radiotracers that bind with sub-nanomolar affinity to the dopamine D3 receptor (D_3_R). In spite of their similar D_3_ affinities, the two PET ligands display very different properties for labeling the D_3_R in vivo: [^18^F]Fallypride is capable of binding to D_3_R under “baseline” conditions, whereas [^18^F]FTP requires the depletion of synaptic dopamine in order to image the receptor in vivo. These data suggest that [^18^F]Fallypride is able to compete with synaptic dopamine for binding to the D_3_R, whereas [^18^F]FTP is not. The goal of this study was to conduct a series of docking and molecular dynamic simulation studies to identify differences in the ability of each molecule to interact with the D_3_R that could explain these differences with respect to competition with synaptic dopamine. Competition studies measuring the ability of each ligand to compete with dopamine in the β-arrestin assay were also conducted. The results of the in silico studies indicate that FTP has a weaker interaction with the orthosteric binding site of the D_3_R versus that of Fallypride. The results of the in silico studies were also consistent with the IC50 values of each compound in the dopamine β-arrestin competition assays. The results of this study indicate that in silico methods may be able to predict the ability of a small molecule to compete with synaptic dopamine for binding to the D_3_R.

## 1. Introduction

Positron emission tomography (PET) is an in vivo, molecular imaging technique capable of providing information on the disease-associated alteration of neurotransmitter function in the living human brain. A neurotransmitter system that has drawn attention in PET imaging studies is the dopaminergic (DAergic) system, and numerous studies can be found in the literature on PET imaging studies of both presynaptic and postsynaptic DAergic function [1,2,3]. This can be attributed to the widely recognized role of the DAergic system in a variety of neurological and neuropsychiatric disorders, including schizophrenia, substance abuse, and Parkinson’s disease (PD) [4,5]. Within the postsynaptic receptors, the D2-family of receptors have been studied in great detail. Members of this receptor family include the D2 (D_2_R; both long and short isoforms), D3 (D_3_R), and D4 (D_4_R) receptors. Although it has been possible to develop radiotracers selective for the D_4_R versus D_2_R and D_3_R [6,7], PET imaging studies of the other members of this family have been conducted using radiotracers that bind with high affinity to both D_2_R and D_3_R, and low affinity for the D_4_R [4,8]. Examples of PET radiotracers falling into this category include [^11^C]raclopride, [^18^F]Fallypride, and [^11^C]PHNO [4,8].

Previous studies from our group have shown that there is a differential distribution of D_2_R and D_3_R in the human brain. For example, the D_2_R is expressed in high density in the caudate, putamen, and nucleus accumbens; however, the density of this receptor in extrastriatal regions, such as the thalamus and substantia nigra, is very low, with ~10% in the striatal regions [9]. Although the D_3_R is expressed in lower density in the caudate, putamen, and nucleus accumbens (~half the density of D_2_R), there is a similar density of this receptor in the striatal regions, thalamus, and nucleus accumbens [9]. Furthermore, we have shown that the D_3_R is an excellent receptor for studying the loss of the nigrostriatal dopaminergic system in PD. There is a reduction in D_3_R in the substantia nigra of postmortem brain samples of PD brain, which is consistent with the loss of the cell bodies of dopamine neurons projecting to the striatal regions. Within the striatum, we observed a dramatic increase in D_3_R density in the caudate, putamen, and nucleus accumbens, which is consistent with denervation sensitization due to the loss of dopamine terminals [10]. Interestingly, no change in the density of D_2_R was observed in the caudate, putamen and, nucleus accumbens of PD brains.

These studies indicate that a D_3_-selective PET radiotracer would be very useful in studying the temporal changes in DAergic function that occur during the disease progression of PD. Furthermore, since the D_3_ receptor has been shown to play a prominent role in other neuropsychiatric disorders, such as substance abuse [11,12,13], a D_3_-selective PET radiotracer is expected to be valuable in a variety of PET imaging studies.

[^18^F]Fluortriopride ([^18^F]FTP) is a PET radiotracer having a high affinity for D_3_R (0.17 nM) and a very low affinity for the D_2_R (~28 nM; selectivity ratio of ~160) [14,15]. PET imaging studies in nonhuman primates revealed that under baseline conditions, [^18^F]FTP displayed no difference in radiotracer uptake between the caudate, putamen, thalamus, and cerebellum. The cerebellum is a reference region for PET imaging studies since it is devoid of dopamine receptors. However, pretreatment with lorazepam to reduce synaptic dopamine levels resulted in a higher uptake of [^18^F]FTP in the caudate, putamen, and thalamus and no change in uptake in the cerebellum [16]. These data suggest that [^18^F]FTP is not able to compete with synaptic dopamine levels under baseline conditions, but it is able to do so once dopamine is reduced via potentiation of the GABAergic system. Interestingly, [^18^F]Fallypride, a non-selective PET radiotracer having a similar D_3_ affinity as that of [^18^F]FTP, is able to image D_3_R in the thalamus under baseline conditions [17]. Pretreatment with amphetamine to cause an elevation in synaptic dopamine levels resulted in only a 7–20% reduction of [^18^F]Fallypride binding in the thalamus [18,19]. These studies demonstrated that [^18^F]Fallypride can compete with synaptic dopamine for binding to thalamic D_3_R much better than [^18^F]FTP, even though they have very similar affinities for the D_3_R.

**Figure 1:**
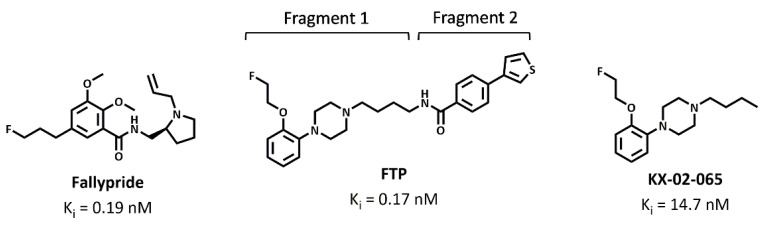
Structures and binding affinity of Fallypride, Fluortriopride (FTP), and KX-02-065 for human D_3_R [4,20].

The goal of this study was to conduct a series of computational chemistry methods to compare the interaction of these two radiotracers at the D_3_R. We also used a β-arrestin assay to directly compare the ability of each compound (i.e., FTP and Fallypride) to compete with synaptic dopamine for binding to the D_3_R. Studies were also performed on the KX-02-065, which is the fragment of FTP that binds to the orthosteric binding site of the D_3_R (Figure 1). The results of this study provide insight into the factors governing D_3_-selectivity and the ability to compete with dopamine for binding to the D_3_R. This information should facilitate the development of D_3_-selective PET radiotracers capable of imaging this receptor in vivo under baseline conditions.

## 2. Materials and Methods

### 2.1. D_3_ Ligands

Fallypride was purchased from ABX (ABX advanced biochemical compounds GmbH, Radeberg, Germany). FTP and KX-02-065 were synthesized as per the previously reported methods [20].

### 2.2. β-Arrestin Recruitment Assay

A PathHunterTM β-arrestin recruitment assay kit and the Chinese hamster ovary (CHO-K1) cell line were purchased from DiscoverX (Fremont, CA, USA). CHO-K1 cells that overexpressed the human D_3_ receptor were cultured in the AssayComplete^TM^ cell culture kit 107. Cells were seeded at a density of 25,000 cells per well of a 96-well plate and incubated at 5% CO_2_ at 37 ℃. Two days later, compounds were dissolved in DMSO, and an 11-point serial dilution was performed in phosphate-buffered saline (PBS). The compounds were added to the cells, and the tubes were incubated for 30 min at 5% CO_2_ at 37 ℃. The cells were then treated with 30 nM (EC80) of dopamine, and the plate was incubated for an additional 90 min. A PathHunterTM detection reagent was added to each well, and the plate was incubated for 80 min at room temperature in the dark. The chemiluminescent signal was measured by the PerkinElmer Enspire plate reader (PerkinElmer, Boston, MA, USA). The data were analyzed by Prism using non-linear regression analysis.

### 2.3. Molecular Docking

The molecular docking studies were performed using the previously reported methods [21]. Fallypride, FTP, and KX-02-065 structures were drawn using the ChemDraw Professional 15.1 (PerkinElmer Informatics, Inc., Waltham, MA, USA). The pyrrolidine ring of Fallypride, the piperazine ring of FTP, and KX-02-065 are expected to be protonated at the physiological pH. Therefore, the nitrogen of the pyrrolidine ring of Fallypride, the nitrogen proximal to the amide bond of the piperazine ring of FTP, and KX-02-065 were protonated. The structures were then imported to the Chem3D Ultra 15.1 (PerkinElmer Informatics, Inc., Waltham, MA, USA) and minimized using MMFF94 force field calculations to prepare for the molecular docking studies. The molecular docking studies were performed via the AutoDock 4.2 [22] plugin on PyMOL (www.pymol.org) (accessed on 25 March 2021). The X-ray structure of D_3_R (PDB ID 3PBL, Resolution 2.89 Å) was obtained from the RCSB Protein Data Bank (www.rcsb.org) (accessed on 25 March 2021). Water molecules and other heteroatoms were removed from the structure, followed by adding polar hydrogens. Nonpolar hydrogens were removed from every compound. A grid box with a dimension of 30 × 30 × 28.2 Å^3^ was applied to the D_3_R X-ray structures covering orthosteric and secondary binding sites. The Lamarckian Genetic Algorithm with a maximum of 2,500,000 energy evaluations was used to calculate 100 D_3_R-ligand binding poses for each compound. The D_3_R−ligand complex that reproduced the crystallographic ligand binding pose and had a good docking score was reported for each compound.

### 2.4. Molecular Dynamics Simulation

#### 2.4.1. Building Protein-Ligand Complex

The CHARMM-GUI web server [23] was used for molecular dynamics simulation (MDS) preparation. The topology and parameter files of protonated Fallypride, FTP, and KX-02-065 were generated by the Ligand Reader and Modeler module [24,25] using the CHARMM General Force Field (CGenFF). The Bilayer Membrane Builder [26,27] module was used for building the MDS system. The protein-ligand complexes generated from the docking studies were aligned to the D_3_R structure (PDB ID: 3PBL) that was obtained from the Orientations of Protein in Membranes (OPM) database [28], and the POPC membrane was placed by using the OPM D_3_R model. The protein, ligand, and membrane complex were solvated in a TIP3P water-box with a volume of 80 × 80 × 112 Å^3^, and then Monte-Carlo sampling was used to add 0.15M NaCl for charge neutralization. The parameters of ligands were converted from CGenFF to General Amber Force Field 2 (GAFF2). Then, GAFF2 for ligand, FF19SB force field for protein, and Amber Lipid17 force field for POPC membrane were used for further performing MDS.

#### 2.4.2. Equilibration and Production Simulations

The MDS studies were performed via the Amber18 [29] on the high-performance computing (HPC) cluster at the Center for Biomedical Image Computing and Analytics at the University of Pennsylvania. The input files of system minimization, 6-step equilibration, and production run for MDS were generated from the last step of the Membrane Builder [26,27] on the CHARMM-GUI web server [23]. The periodic boundary conditions were used for the MDS studies. The SHAKE algorithm was used to constrain bonds involving hydrogen atoms. Energy minimization of 5000 steps was implemented. Then, the minimized system was heated in a 2-step NVT ensemble with constant volume at 310 K for 500 ps with a time step of 1 fs in each step. Then, the system was equilibrated in a 4-step NPT ensemble at 310 K and 1 atm for a total of 3500 ps (500 ps with 1 fs time step at the first step of NPT ensemble, following by 1000 ps with 2 fs time step at the second to the fourth steps of NPT ensemble). The system minimization and equilibration simulations were performed using the pmemd.MPI in Amber18 [29] on 40 CPUs. Five copies of the production simulations were performed for 200 ns with a time step of 2 fs in each copy and using the pmemd.cuda Amber18 [29] on NVIDIA P100 GPU.

#### 2.4.3. Molecular Dynamics Simulation Analysis

For further MDS analysis, 50 to 200 ns of each production simulation was used. The ParmEd module in the AmberTool18 [29] was performed to remove the solvent, membrane, and ions from the topology of the MDS system, and it generated the respective topology for protein, ligand, and protein-ligand complexes for MM/GBSA calculation. A total of 7500 frames (1500 frames of every 5 production simulation copies) were processed by using the single trajectory approach of MM/GBSA in the AmberTool18 [29] for calculating the free energy of binding. The per-residue free energy decomposition of each residue in the binding pocket and the pair-wise free energy decomposition of ligand to each residue in the binding site were also calculated. The interactions between ligand and protein in the production simulations were computed by using the Getcontacts script tool (https://getcontacts.github.io/) (accessed on 25 March 2021). The MDAnalysis [30,31] python toolkit was used for distance analysis.

## 3. Results

### 3.1. β-Arrestin Recruitment Assays

**Figure 2:**
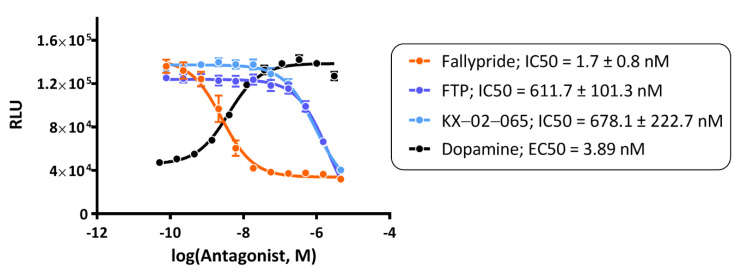
Fallypride, FTP, and KX-02-065 were investigated as antagonists for D_3_ by β-arrestin recruitment assay. For antagonist mode, functional assays were performed in the presence of 30 nM dopamine. The graph represented a single experiment, and the IC50 value was represented of the mean ± sd of three to four individual experiments.

Fallypride, FTP, and KX-02-065 were tested for their functional antagonism of dopamine at the D_3_R using the β-arrestin recruitment assay. The EC50 value of dopamine in this assay was 3.89 nM. All compounds were tested in the antagonist mode in the presence of 30 nM dopamine. Fallypride had the highest potency in this assay (IC50 = 1.7 ± 0.8 nM) (Figure 2). In contrast, FTP had a much lower potency than Fallypride (IC50 = 611.7 ± 101.3 nM) and was similar to that of KX-02-065 (IC50 = 678.1 ± 222.7 nM) (Figure 2).

### 3.2. Molecular Docking

**Figure 3:**
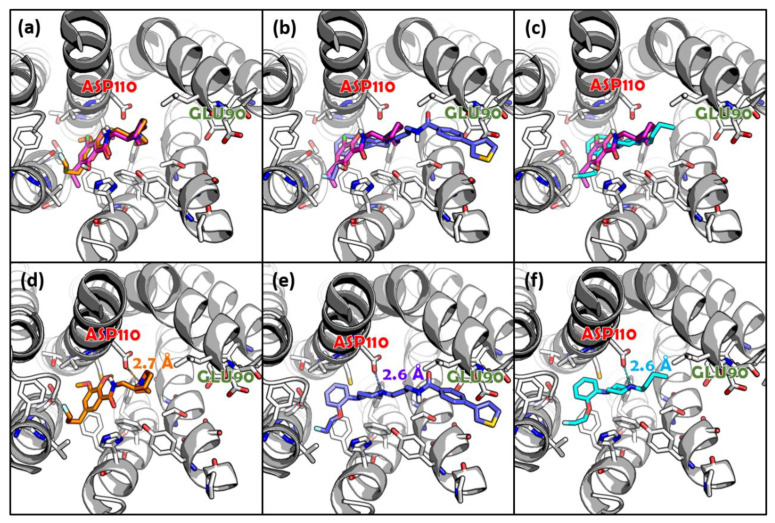
Docking studies of (**a**,**d**) Fallypride, (**b**,**e**) FTP, and (**c**,**f**) KX-02-065 to D_3_R. Alignment of docking pose for (**a**) Fallypride, (**b**) FTP, and (**c**) KX-02-065 to the crystallographic ligand (eticlopride) in D_3_R. The individual binding poses and the distance between the protonated hydrogen of (**d**) Fallypride, (**e**) FTP, and (**f**) KX-02-065. Color label: purple (eticlopride); orange (Fallypride); blue (FTP); cyan (KX-02-065).

The docking poses of Fallypride, FTP, and KX-02-065 were reproduced using the geometry of the crystallographic data with eticlopride in the orthosteric binding site (Figure 3a–c). The root-mean-square distance (RMSD) between eticlopride and Fallypride, FTP fragment 1, and KX-02-065 were 4.4 Å, 5.8 Å, and 4.2 Å, respectively. FTP’s selected binding pose (predicted binding energy = −9.15 kcal/mol) showed the best docking score compared to Fallypride (predicted binding energy = −7.71 kcal/mol) and KX-02-065 (predicted binding energy = −6.10 kcal/mol). All three ligands were observed forming a salt bridge with similar distances between ASP110 and the protonated nitrogen of each compound (2.7 Å for Fallypride, and 2. 6 Å for both FTP and KX-02-065; Figure 3d–f).

### 3.3. Molecular Dynamics Simulation

To evaluate the stability of Fallypride, FTP, and KX-02-065 in the D_3_R binding pocket, the RMSD of each compound over 50–200 ns in 5 copies of the production MDS were calculated by using the first frame (0 ns) of the production run as the reference position. The averaged RMSD over the simulation time of three compounds were similar; Fallypride (2.08 ± 0.33 Å) had the least amount of motion in the binding pocket and the lowest standard deviation of RMSD as compared to FTP (2.11 ± 0.43 Å) and KX-02-065 (2.15 ± 0.96 Å). FTP fragment 1 (1.74 ± 0.26 Å) displayed little movement in the orthosteric binding site, whereas a higher movement of fragment 2 (6.24 ± 0.96 Å) was observed in the secondary binding site.

The MM/GBSA free energy of binding, which is used to predict the binding potency of each compound, is shown in Table 1. The best predicted binding potency among the three compounds was FTP, followed by Fallypride and KX-02-065. The lowest van der Waals and electrostatic energy contribution were observed in FTP and Fallypride, respectively.

**Figure 4:**
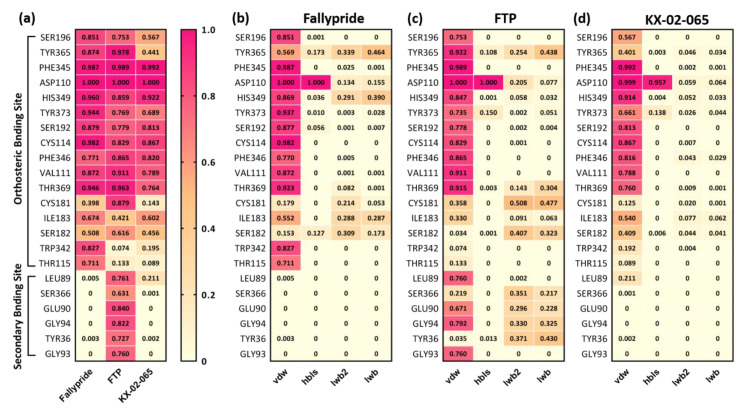
(**a**) The summation of all types of the frequency of contacts between the residues in the binding pocket and Fallypride, FTP, and KX-02-065. The frequency of van der Waals (vdW), ligand-sidechain hydrogen bonds (hbls), ligand water-mediated hydrogen bond (lwb), and ligand extended water-mediated hydrogen bond (lwb2) interactions between sidechains and (**b**) Fallypride, (**c**) FTP, and (**d**) KX-02-065. The summation of all types of the frequency of contacts between any residue and any ligand higher than 0.6 is shown in the figure.

**Figure 5:**
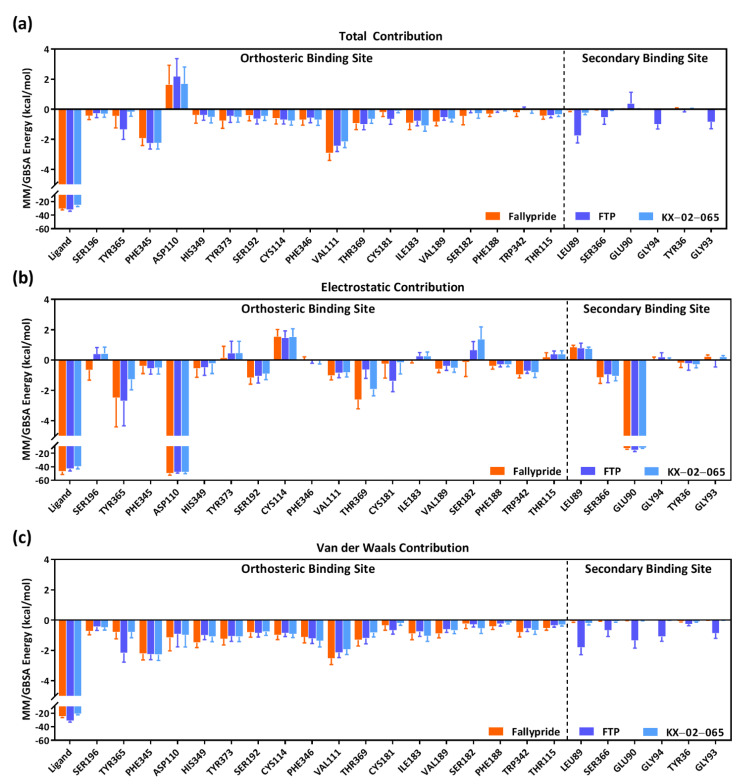
(**a**) Total MM/GBSA binding free energy (kcal/mol) contribution of each residue in the binding pocket, (**b**) MM/GBSA electrostatic (kcal/mol) contribution of each residue in the binding pocket, and (**c**) MM/GBSA van der Waals (kcal/mol) contribution of each residue in the binding pocket.

**Figure 6:**
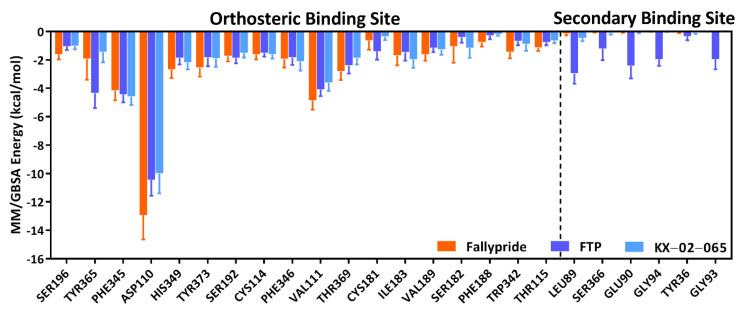
Total MM/GBSA binding free energy (kcal/mol) contribution of ligands to residue pairs.

In order to further investigate the interactions of the ligands within the binding pocket of D_3_R, the frequency of contacts (Figure 4) and decomposition energies (Figure 5 and Figure 6) for each ligand and the different residues in the orthosteric and secondary binding sites were calculated. Figure 4a shows a summary of the contact frequencies between each ligand and the different residues in both the orthosteric and secondary binding pockets. Overall, all three compounds formed high-frequency interactions with the residues in the orthosteric binding site (frequency of contact >0.6 for ASP110, VAL111, CYS114, SER192, PHE345, PHE346, HIS349, THR369, and TYR373); only FTP formed high-frequency interactions with the residues in the secondary binding site (frequency of contact >0.6 for TYR36, LEU89, GLU90, GLY93, GLY94, and SER366). The protonated nitrogen of Fallypride and FTP continually formed hydrogen bonds with ASP110 during the entire MDS study (frequency of contact = 1.0). The frequency of the hydrogen bond formed between ASP110 and the protonated nitrogen of KX-02-065 (frequency of contact = 0.957) was slightly lower than that of FTP and Fallypride (Figure 4b–d). The protein–ligand interactions that formed with the residues in the orthosteric binding site for Fallypride, FTP, and KX-02-065 were mostly van der Waals interactions. In the secondary binding site, only FTP formed high-frequency van der Waals interactions with LEU89 (0.760), GLU90 (0.671), GLY94 (0.792), and GLY93 (0.760). An intermediate frequency of contacts (0.2–0.4) of the ligand water-mediated hydrogen bond and ligand extended water-mediated hydrogen bond was observed between FTP and SER366, GLU90, GLY94, and TYR36 (Figure 4c).

Figure 5 and Table S1–S3 show the contribution of decomposition energies computed from MM/GBSA to the system’s binding free energy of each residue in the D_3_R binding pocket. Approximately 50–60% of the total binding free energy was attributed to the total decomposition energy of the ligand in all the D_3_R–ligand complexes: 53.41% of D_3_R–Fallypride complex (total decomposition energy of ligand = −24.94 ± 1.67 kcal/mol vs. total binding free energy = −46.70 ± 4.38 kcal/mol), 60.18% of D_3_R–FTP complex (total decomposition energy of ligand = −31.15 ± 2.02 kcal/mol vs. total binding free energy = −51.76 ± 4.43 kcal/mol), and 54.12% of D_3_R–KX-02-065 complex (total decomposition energy of ligand = −20.97 ± 1.49 kcal/mol vs. total binding free energy = −38.75 ± 4.19 kcal/mol). There were no differences in the contribution of binding in the orthosteric binding site to the total binding free energy with Fallypride, FTP, and KX-02-065. The contribution to the total binding free energy of residues in the secondary binding site were all close to zero for D_3_R–Fallypride (−0.09 to 0.06 kcal/mol) and D_3_R–KX-02-065 (−0.24 to 0.03 kcal/mol) complex, whereas a small portion of contribution was observed in the D_3_R–FTP complex (−1.77 to 0.38 kcal/mol). The decomposition electrostatic energy of ligand, ASP110, and GLU90 were lower than −12 kcal/mol in the three D_3_R–ligand complexes, representing that at least 13% of the electrostatic energy can be attributed to this interaction (Figure 5b). The electrostatic energy of ASP110 in the Fallypride system (−49.82 ± 2.54 kcal/mol) was significantly lower than in the FTP (−47.55 ± 1.79 kcal/mol) and KX-02-065 (−47.60 ± 2.46 kcal/mol) systems, and GLU90 in the FTP system (−15.66 ± 1.96 kcal/mol) was significantly lower than in Fallypride (−12.98 ± 1.17 kcal/mol) and KX-02-065 (−12.14 ± 0.94 kcal/mol). The van der Waals energy contribution to the binding free energy of each residue was similar to the total energy contribution of each residue. A significant amount of van der Waals energy was contributed from ligands as compared to the protein residues in all D_3_R–Fallypride (−24.94 ± 1.67 kcal/mol), D_3_R–FTP (−31.15 ± 2.02 kcal/mol), and D_3_R–KX-02-065 (−20.97 ± 1.49 kcal/mol) complexes; the D_3_R–FTP complex (−1.81 to −0.26 kcal/mol) was the only complex to show the van der Waals energy contribution from the secondary binding site (Figure 5c).

The free energy of interaction with ASP110 represented a major contribution to the total binding free energy in the orthosteric binding site for all three D_3_R–ligand complexes (Figure 6 and Table S4). The free energy of ASP110 to Fallypride interaction (−12.93 ± 1.71 kcal/mol) was significantly lower than the interaction of ASP110 to FTP (−10.46 ± 1.11 kcal/mol) and KX-02-065 (−10.01 ± 1.40 kcal/mol). The free energy of interaction with residues in the secondary binding site was close to zero for Fallypride (−0.14 to 0.00 kcal/mol) and KX-02-065 (−0.47 to 0.00 kcal/mol); only the D_3_R–FTP complex (−2.96 to −0.35 kcal/mol) showed a ligand-to-protein interaction that contributed to the total binding free energy in the secondary binding site.

## 4. Discussion

[^18^F]Fallypride and [^18^F]FTP are two different PET radiotracers that bind with sub-nM affinity to the D_3_R [32,33]. The docking results of Fallypride reproduced the binding geometry of eticlopride in the X-ray crystal structure. Taking the docking pose to the MDS studies revealed that Fallypride formed extensive interactions with the orthosteric binding site, suggesting that the sub-nM binding affinity for Fallypride can be attributed to this interaction with the D_3_R.

In previous in vitro and in silico studies, it has been reported that FTP’s high-affinity binding at the D_3_R is attributed to its bitopic properties by interacting with both the orthosteric and secondary binding sites [20]. In our study, docking and MDS results of FTP reproduced this bitopic binding mode: FTP fragment 1 interacted with the orthosteric binding site and FTP fragment 2 interacted with the secondary binding site in the D_3_R. The RMSD of FTP fragment 1 and fragment 2 in the D_3_R–FTP MDS system were 1.74 ± 0.26 Å and 6.24 ± 0.96 Å, and the RMSD of the FTP fragment 1 alone in the D_3_R–KX-02-065 complex was 2.15 ± 0.96 Å. This indicated that with the interaction of the secondary binding site, FTP fragment 2 enhanced the stability of the orthosteric binding site interaction for FTP fragment 1. This is also supported by the summation of the ligand-residue pair contributions to the total free energy of binding in the orthosteric and the secondary binding sites. The contribution to the free energy of binding of the ligand to the orthosteric binding site was lower in the D_3_R–FTP complex as compared with the D_3_R–KX-02-065 complex.

The potencies of the predicted binding free energy for Fallypride and FTP were consistent with the measured in vitro binding affinity. In the D_3_R–FTP complex, ~20% of the binding free energy was contributed by FTP interacting with the secondary binding site, and ~80% was contributed by interacting with the orthosteric binding site. However, in the D_3_R–Fallypride complex, greater than 99% of the binding free energy was contributed by interacting with the orthosteric binding site. Although both ligands have similar binding affinities in D_3_R, these findings indicate that the binding modes for FTP and Fallypride to D_3_R are very different.

In previous PET imaging studies, [^18^F]Fallypride and [^18^F]FTP display very different properties for imaging the D_3_R in vivo. [^18^F]Fallypride is capable of binding to D_3_R under baseline conditions based on its high uptake in brain regions with a high density of D_3_R. On the other hand, [^18^F]FTP requires the depletion of synaptic dopamine in order to image the receptor in vivo [4]. These data indicate that [^18^F]Fallypride is able to compete with synaptic dopamine for binding to the D_3_R in vivo, whereas [^18^F]FTP is not. Our results of β-arrestin recruitment assays were consistent with these in vivo studies. That is, Fallypride had an IC50 of ~2 nM, indicating its strong ability to compete with endogenous dopamine for the D_3_R. In contrast, the IC50 of FTP showed a low functional potency for D_3_R (IC50 > 500 nM), suggesting that FTP is not able to compete with synaptic dopamine for the D_3_R. The similar potency of FTP and KX-02-065 in the β-arrestin assay (IC50 > 500 nM) suggests that the potency of the ligand (or in the case of FTP, the fragment of the ligand) that interacts with the orthosteric binding site determines its ability to compete with endogenous dopamine for binding to the D_3_R.

In the MDS studies, the rank of the ASP110–ligand pairs’ contribution to the total binding free energy was consistent with the measurement potency of IC50 from β-arrestin recruitment assays. The free energy of the ASP110–Fallypride pair was significantly lower than ASP110–FTP and ASP110–KX-02-065 pairs, whereas no difference was observed between ASP110–FTP and ASP110–KX-02-065 pairs. It has been reported that the electrostatic interaction of ASP110 to ligands in the D_3_R plays an important role in the compound binding affinity to D_3_R [20,34]. Our results also showed a high electrostatic contribution of ASP110 and high binding free energy contribution for each ASP110–ligand pair, indicating the contact formation between ligand and ASP110 is the key interaction in D_3_R. These results indicate that in silico measurements of the free energy of binding for a ligand–ASP110 pair in the orthosteric binding site may be able to predict the ability of a small molecule to compete with synaptic dopamine for binding to the D_3_R in vivo.

The results of in silico approaches performed in this study were consistent with the results of in vitro measurements and also supported the in vivo behavior differences between Fallypride and FTP for the D_3_R. Although the MM/GBSA free energy calculation has widely used for predicting free energy of binding in drug design for multiple targets, the prediction is highly dependent on the setting of parameters for the MDS system [35]. Therefore, validation of our current modeling approaches for the D_3_R in a larger scale of diverse compound libraries will be explored in the future.

## 5. Conclusions

In summary, our studies have provided key information to explain the in vivo behavior of two different PET radiotracers that have a high affinity for the D_3_R. Fallypride has a sub-nM affinity for the D_3_R and is able to compete with synaptic dopamine for binding to the receptor because its primary contacts are located within the orthosteric binding site. This was confirmed by its low RMSD in the MDS studies, low free energy of binding in the orthosteric binding site, and strong interaction with ASP110. On the other hand, the high binding affinity of FTP is attributed to its interaction with both the orthosteric and secondary binding sites in the D_3_R. The fragment of FTP interacting with the orthosteric site has a higher RMSD value in MDS simulation studies and worse free energy of interaction with ASP110 compared to Fallypride. The interaction of FTP with the secondary binding site is responsible for its lower affinity for the D_2_R and high selectivity for the D_3_R, whereas Fallypride has a high affinity for both D_2_R and D_3_R because it does not interact with the secondary binding sites in each receptor. A key step in identifying a D_3_R-selective radiotracer that is capable of competing with endogenous dopamine for the D_3_R would be to identify a small molecule that has the optimal orthosteric binding properties of Fallypride and a suitable interaction with the secondary binding site, as observed with FTP. A β-arrestin competition assay is also a useful tool for predicting the ability of a small molecule to compete with dopamine for binding to the D_3_R.

## Figures and Tables

**Table 1:** Summary of MM/GBSA binding free energy (kcal/mol).

	Delta Total	Van der Waals	Electrostatic
Fallypride	−46.70 ± 4.38	−49.89 ± 3.63	−94.13 ± 9.41
FTP	−51.76 ± 4.43	−62.30 ± 4.91	−85.64 ± 9.18
KX-02-065	−38.75 ± 4.19	−41.95 ± 3.28	−79.38 ± 9.22

## Data Availability

The article contains complete data used to support the findings of this study.

## Supplementary Materials

The following are available online at https://www.mdpi.com/article/10.3390/biom11040529/s1, Table S1: Total MM/GBSA energy (kcal/mol) contribution of residues in the binding pocket, Table S2: Van der Waals MM/GBSA energy (kcal/mol) contribution of residues in the binding pocket, Table S3: Electrostatic MM/GBSA energy (kcal/mol) contribution of residues in the binding pocket, and Table S4: Ligand to residue pair MM/GBSA energy (kcal/mol) contribution.
