# Supplementary material for: Interaction of Ligands for PET with the Dopamine D3 Receptor: In Silico and In Vitro Methods
Source: Biomolecules. 2021 Apr 2;11(4):529. doi: 10.3390/biom11040529 (PMC8065765; doi:10.3390/biom11040529)
Supplement: Supplementary file 1 [file biomolecules-11-00529-s001.pdf]

biomolecules

MDPI

Article

Interaction of PET Radioligands with the Dopamine D3 Receptor: In Silico and In Vitro Methods

Chia-Ju Hsieh [1], Aladdin Riad [1], Ji Youn Lee [1], Kristoffer Sahlholm [2], Kuiying Xu [1], Robert R. Luedtke [3], Robert H. Mach [1,*]

[1] Division of Nuclear Medicine and Clinical Molecular Imaging, Department of Radiology, Perelman School of Medicine, University of Pennsylvania, Philadelphia, PA 19104, USA; chiahs@pennmedicine.upenn.edu (C.-J.H.); Aladdin.Riad@pennmedicine.upenn.edu (A.R.); JiYoun.Lee@Pennmedicine.upenn.edu (J.Y.L.); kuxu@pennmedicine.upenn.edu (K.X.)

[2] Department of Integrative Medical Biology, Wallenberg Center for Molecular Medicine, Umea University, Umea 901 87, Sweden; kristoffer.sahlholm@ki.se

[3] Department of Pharmacology and Neuroscience, University of North Texas Health Science Center-Fort Worth, Texas, TE 76107, USA; Robert.Luedtke@unthsc.edu

* Correspondence: rmach@pennmedicine.upenn.edu

Table S1. Total MM/GBSA energy (kcal/mol) contribution of residues in the binding pocket.

Residue	Fallypride	FTP	KX-02-065
Ligand	−30.29 ± 2.04	−32.21 ± 1.97	−25.44 ± 1.90
	Orthosteric binding Site		
SER196	−0.43 ± 0.26	−0.28 ± 0.27	−0.31 ± 0.22
TYR365	−0.45 ± 0.79	−1.35 ± 0.65	−0.17 ± 0.30
PHE345	−1.94 ± 0.47	−2.27 ± 0.38	−2.24 ± 0.41
ASP110	1.65 ± 1.28	2.18 ± 1.19	1.70 ± 1.11
HIS349	−0.39 ± 0.53	−0.39 ± 0.34	−0.53 ± 0.38
TYR373	−0.76 ± 0.50	−0.46 ± 0.41	−0.53 ± 0.33
SER192	−0.41 ± 0.34	−0.64 ± 0.34	−0.46 ± 0.29
CYS114	−0.62 ± 0.36	−0.71 ± 0.27	−0.76 ± 0.29
PHE346	−0.70 ± 0.36	−0.58 ± 0.32	−0.69 ± 0.38
VAL111	−2.93 ± 0.47	−2.45 ± 0.36	−2.15 ± 0.40
THR369	−0.95 ± 0.40	−1.01 ± 0.35	−0.65 ± 0.29
CYS181	−0.20 ± 0.29	−0.65 ± 0.36	−0.12 ± 0.11
ILE183	−0.93 ± 0.42	−0.79 ± 0.31	−1.11 ± 0.35
VAL189	−0.82 ± 0.28	−0.54 ± 0.19	−0.63 ± 0.21
SER182	−0.45 ± 0.58	−0.06 ± 0.17	−0.28 ± 0.31
PHE188	−0.31 ± 0.18	−0.09 ± 0.10	−0.05 ± 0.05
TRP342	−0.23 ± 0.25	0.04 ± 0.11	−0.06 ± 0.20
THR115	−0.45 ± 0.21	−0.41 ± 0.15	−0.35 ± 0.13
	Secondary binding Site		
LEU89	−0.09 ± 0.07	−1.77 ± 0.46	−0.24 ± 0.12
SER366	−0.01 ± 0.02	−0.55 ± 0.45	−0.03 ± 0.05
GLU90	0.00 ± 0.02	0.38 ± 0.74	0.01 ± 0.03
GLY94	0.01 ± 0.00	−1.00 ± 0.31	0.00 ± 0.00
TYR36	0.06 ± 0.03	−0.04 ± 0.13	0.03 ± 0.03
GLY93	0.01 ± 0.00	−0.85 ± 0.44	0.01 ± 0.00

Table S2. Van der Waals MM/GBSA energy (kcal/mol) contribution of residues in the binding pocket.

Residue	Fallypride	FTP	KX-02-065
Ligand	−24.94 ± 1.67	−31.15 ± 2.02	−20.97 ± 1.49
**Orthosteric binding Site**			
SER196	−0.73 ± 0.24	−0.45 ± 0.23	−0.48 ± 0.18
TYR365	−0.79 ± 0.44	−2.18 ± 0.59	−0.79 ± 0.38
PHE345	−2.22 ± 0.40	−2.27 ± 0.35	−2.29 ± 0.38
ASP110	−1.17 ± 0.86	−0.92 ± 0.84	−0.99 ± 0.78
HIS349	−1.49 ± 0.32	−1.00 ± 0.29	−1.10 ± 0.32
TYR373	−1.23 ± 0.40	−1.07 ± 0.34	−1.10 ± 0.32
SER192	−0.82 ± 0.30	−0.85 ± 0.27	−0.77 ± 0.24
CYS114	−0.99 ± 0.30	−0.85 ± 0.23	−0.91 ± 0.25
PHE346	−1.12 ± 0.38	−1.22 ± 0.32	−1.36 ± 0.41
VAL111	−2.53 ± 0.41	−2.15 ± 0.33	−1.93 ± 0.35
THR369	−1.32 ± 0.39	−1.21 ± 0.36	−0.83 ± 0.28
CYS181	−0.36 ± 0.31	−0.68 ± 0.25	−0.22 ± 0.13
ILE183	−0.89 ± 0.40	−0.77 ± 0.30	−1.06 ± 0.34
VAL189	−0.89 ± 0.28	−0.61 ± 0.20	−0.69 ± 0.20
SER182	−0.26 ± 0.30	−0.28 ± 0.17	−0.55 ± 0.32
PHE188	−0.42 ± 0.19	−0.25 ± 0.14	−0.18 ± 0.07
TRP342	−0.81 ± 0.30	−0.56 ± 0.19	−0.67 ± 0.27
THR115	−0.53 ± 0.14	−0.36 ± 0.10	−0.31 ± 0.09
**Secondary binding Site**			
LEU89	−0.09 ± 0.06	−1.81 ± 0.47	−0.21 ± 0.11
SER366	−0.06 ± 0.02	−0.68 ± 0.40	−0.08 ± 0.06
GLU90	−0.02 ± 0.01	−1.34 ± 0.51	−0.03 ± 0.01
GLY94	−0.01 ± 0.00	−1.10 ± 0.30	−0.01 ± 0.01
TYR36	−0.08 ± 0.04	−0.26 ± 0.11	−0.10 ± 0.04
GLY93	0.00 ± 0.00	−0.87 ± 0.33	0.00 ± 0.00

Table S3. Electrostatic MM/GBSA energy (kcal/mol) contribution of residues in the binding pocket.

Residue	Fallypride	FTP	KX-02-065
Ligand	−47.07 ± 4.31	−42.82 ± 3.43	−39.69 ± 3.47
**Orthosteric binding Site**			
SER196	−0.66 ± 0.66	0.40 ± 0.42	0.43 ± 0.42
TYR365	−2.49 ± 1.91	−2.70 ± 1.63	−1.28 ± 0.68
PHE345	−0.41 ± 0.49	−0.57 ± 0.35	−0.51 ± 0.40
ASP110	−49.82 ± 2.54	−47.55 ± 1.79	−47.60 ± 2.46
HIS349	−0.56 ± 0.57	−0.49 ± 0.51	−0.25 ± 0.64
TYR373	0.16 ± 0.75	0.46 ± 0.79	0.46 ± 0.77
SER192	−1.16 ± 0.42	−1.07 ± 0.45	−0.90 ± 0.39
CYS114	1.56 ± 0.46	1.46 ± 0.46	1.53 ± 0.53
PHE346	0.02 ± 0.19	−0.04 ± 0.18	−0.05 ± 0.21
VAL111	−1.02 ± 0.28	−0.87 ± 0.28	−0.82 ± 0.29
THR369	−2.62 ± 0.60	−0.64 ± 0.57	−1.92 ± 0.43
CYS181	−0.25 ± 0.93	−1.38 ± 0.70	−0.18 ± 0.73
ILE183	−0.02 ± 0.17	0.27 ± 0.23	0.26 ± 0.27
VAL189	−0.61 ± 0.21	−0.40 ± 0.28	−0.54 ± 0.27
SER182	−0.14 ± 0.94	0.65 ± 0.56	1.37 ± 0.81
PHE188	−0.42 ± 0.19	−0.30 ± 0.15	−0.30 ± 0.15

TRP342	−0.95 ± 0.22	−0.72 ± 0.15	−0.83 ± 0.32
THR115	0.19 ± 0.29	0.38 ± 0.22	0.39 ± 0.21
**Secondary binding Site**			
LEU89	0.85 ± 0.13	0.80 ± 0.31	0.74 ± 0.11
SER366	−1.14 ± 0.39	−0.96 ± 0.53	−1.05 ± 0.31
GLU90	−12.98 ± 1.17	−15.66 ± 1.96	−12.14 ± 0.94
GLY94	0.05 ± 0.15	0.20 ± 0.28	0.00 ± 0.13
TYR36	−0.20 ± 0.30	−0.24 ± 0.44	−0.30 ± 0.21
GLY93	0.24 ± 0.09	−0.04 ± 0.42	0.21 ± 0.09

Table S4. Ligand to residue pair MM/GBSA energy (kcal/mol) contribution.

Residues	Fallypride	FTP	KX-02-065
**Orthosteric binding Site**			
SER196	−1.62 ± 0.35	−1.06 ± 0.25	−1.02 ± 0.24
TYR365	−1.93 ± 1.47	−4.34 ± 1.05	−1.44 ± 0.73
PHE345	−4.16 ± 0.68	−4.43 ± 0.55	−4.58 ± 0.59
ASP110	−12.93 ± 1.71	−10.46 ± 1.11	−10.01 ± 1.40
HID349	−2.69 ± 0.59	−1.87 ± 0.45	−2.18 ± 0.48
TYR373	−2.55 ± 0.62	−1.83 ± 0.61	−1.91 ± 0.57
SER192	−1.74 ± 0.37	−1.87 ± 0.37	−1.52 ± 0.32
CYS114	−1.64 ± 0.33	−1.50 ± 0.26	−1.62 ± 0.29
PHE346	−1.94 ± 0.59	−1.84 ± 0.51	−2.11 ± 0.65
VAL111	−4.85 ± 0.65	−4.09 ± 0.46	−3.63 ± 0.55
THR369	−2.82 ± 0.59	−2.41 ± 0.56	−1.87 ± 0.46
CYS181	−0.64 ± 0.64	−1.40 ± 0.59	−0.38 ± 0.22
ILE183	−1.70 ± 0.68	−1.47 ± 0.57	−1.96 ± 0.59
VAL189	−1.61 ± 0.45	−1.15 ± 0.30	−1.29 ± 0.35
SER182	−1.05 ± 1.15	−0.41 ± 0.39	−1.16 ± 0.69
PHE188	−0.77 ± 0.29	−0.30 ± 0.24	−0.18 ± 0.11
TRP342	−1.44 ± 0.44	−0.67 ± 0.29	−0.89 ± 0.47
THR115	−1.13 ± 0.23	−0.76 ± 0.21	−0.63 ± 0.19
**Secondary binding Site**			
LEU89	−0.14 ± 0.12	−2.96 ± 0.71	−0.47 ± 0.21
SER366	−0.06 ± 0.02	−1.21 ± 0.80	−0.11 ± 0.11
GLU90	−0.07 ± 0.02	−2.42 ± 0.88	−0.08 ± 0.02
GLY94	−0.01 ± 0.01	−1.96 ± 0.46	−0.01 ± 0.01
TYR36	−0.04 ± 0.07	−0.35 ± 0.26	−0.09 ± 0.07
GLY93	0.00 ± 0.00	−1.95 ± 0.71	0.00 ± 0.00
**Sum of Orthosteric binding Site**	−43.86 ± 0.76	−40.14 ± 0.55	−36.68 ± 0.57
**Sum of Secondary binding Site**	−0.32 ± 0.06	−10.87 ± 0.67	−0.76 ± 0.10
**Total**	−44.18 ± 0.66	−51.01 ± 0.58	−37.44 ± 0.50
